# RNA-Silencing Enzymes Pol IV and Pol V in Maize: More than one Flavor?

**DOI:** 10.1371/journal.pgen.1000736

**Published:** 2009-11-20

**Authors:** Craig S. Pikaard, Sarah Tucker

**Affiliations:** 1 Department of Biology, Indiana University, Bloomington, Indiana, United States of America; 2 Department of Molecular and Cellular Biochemistry, Indiana University, Bloomington, Indiana, United States of America; 3 Department of Biology, Washington University in St. Louis, St. Louis, Missouri, United States of America; The University of North Carolina at Chapel Hill, United States of America

There are some puzzling genetic phenomena in nature that have kept geneticists busily searching for clues for decades. Among genetics' X-files are cases in which alleles of the same gene have the same sequence but very different functional states. Gene behaviors of this sort fall under the heading of “epigenetic phenomena.” Traits attributable to epigenetically altered alleles (epialleles) are often not transmitted between generations as independent, immutable characters, according to the rules that Mendel worked out with his garden peas. Instead, there are cases where alleles can change state if and when they find themselves in the same nucleus as epialleles that are locked into a different functional state. This is the case in paramutation, which describes the situation whereby a silent, paramutagenic allele can influence the expression of an active, paramutable allele of the same gene when the two alleles are exposed to one another within the same genome [1]. The silent allele, which is defined as being paramutagenic, somehow transfers its silent state to the other allele, causing that previously active allele to become silenced, too. Most interestingly, the previously active allele is now stably altered in such a way that it can retain the silenced state through meiosis and future generations. Moreover, the newly silenced allele is paramutagenic itself and will silence compatible paramutable alleles. All of this happens without any changes in the nucleotide sequence at the affected locus. This unusual allelic behavior is the genic analogue of the shape-shifting that occurs among proteins that can convert to a prion state and then cause other proteins of identical amino acid sequence to acquire the altered conformation [2],[3]. How alleles can influence one another to acquire or maintain a paramutated or paramutagenic state is not yet known. However, in this issue of *PLoS Genetics*, two interesting papers from the Hollick (Stonaker et al. [4]) and Chandler (Sidorenko et al. [5]) laboratories provide further evidence that paramutation in maize has an RNA basis. Specifically, the studies suggest that multiple alternative subunits of the plant-specific multi-subunit RNA polymerases, Pol IV and Pol V, first discovered in *Arabidopsis* [6],[7], might assemble into Pol IV or Pol V sub-types that have different roles in paramutation and gene silencing.

## Paramutation and siRNA-Mediated DNA Methylation

Paramutation occurs in animals as well as plants, but was first described in maize in the 1950s based on the behavior of genes that affect pigmentation of the plants and/or seeds. Sidorenko et al. and Stonaker et al. based their studies on the *B1* (booster1) and *Pl1-Rhoades* (purple plant1-Rhoades) genes, both of which are involved in red/purple pigmentation. Certain alleles, such as *B-I* (B-intense) and *Pl1-Rh*, are fully functional and cause dark pigmentation, but alternative epialleles of these genes are silent, paramutagenic and designated with apostrophes (*B'* and *Pl'*). *B-I* and *Pl-Rh* alleles can spontaneously convert to *B'* and *Pl'* epialleles, but it is generally a one-way street; once converted, *B'* and *Pl'* alleles do not readily revert to the active state. Moreover, exposure of a functional allele to a corresponding *B'* or *Pl'* allele silences the active allele and converts it to a *B'* or *Pl'* allele. The predictability of these allelic behaviors and interactions has allowed for genetic screens to find mutants that are defective for the establishment or maintenance of paramutation.

The outcome of the maize genetic screens, to date, is the revelation that paramutation requires components of the 24-nt siRNA-directed DNA methylation pathway defined in considerable detail in *Arabidopsis thaliana* [7]. This pathway is required for the transcriptional silencing of transposable elements, endogenous repeats, and foreign transgenes. The pathway involves two plant-specific DNA-dependent RNA polymerases [8]–[11], now abbreviated as Pol IV and Pol V [12],[13]; the RNA-dependent RNA polymerase, RDR2; the double-stranded RNA endonuclease, DICER-LIKE3 (DCL3); and at least two of the ten Argonaute proteins, AGO4 and AGO6 [7]. Other proteins of the pathway include two putative chromatin remodeling ATPases—CLSY1 and DRD1 [14],[15] —and the de novo cytosine methyltransferase, DRM2 [16]. Pol IV acts early in the pathway, presumably generating transcripts that are then used as templates by RDR2, thereby producing double-stranded RNAs that are cleaved into 24-nt double-stranded siRNAs by DCL3. CLSY1 is thought to assist transcription by Pol IV and/or RDR2 [14]. Independent of siRNA biogenesis, Pol V generates transcripts that overlap loci that are subjected to siRNA-directed DNA methylation [12]. DRD1 is required for the production of these Pol V transcripts [12]. AGO4 can be crosslinked to Pol V transcripts [17], suggesting that structural RNAs synthesized by Pol V act as scaffolds for the recruitment of AGO4-siRNA complexes and the transcriptional silencing machinery, including DRM2. However, the biochemical details of how transcriptional repression is actually established are not yet in hand.

Evidence that proteins of the RNA-directed DNA methylation pathway are required for paramutation first came in 2006 when the gene responsible for the mediator of paramutation 1 (*mop1*) mutation was shown by the Chandler lab to be the maize ortholog of *Arabidopsis RDR2* [18]. Two important papers from the Hollick lab soon followed. First, the required to maintain repression 1 (*rmr1*) gene was shown to encode a putative chromatin remodeling ATPase related to *Arabidopsis CLSY1* and *DRD1* [19]. Next, mutations defining the *rmr6* locus were shown to disrupt shown to disrupt the maize ortholog of *Arabidopsis* NRPD1, the largest subunit of nuclear DNA-dependent RNA polymerase IV [20].

The morphological consequences of *rdr2/mop1* and *nrpd1* disruption are more severe in maize than in *Arabidopsis*. In *Arabidopsis*, *rdr2-* and *nrpd1*-null mutants cause an essentially complete loss of 24-nt siRNAs, loss of siRNA-directed DNA methylation, and derepression of transposons. The *rdr2* and *nrpd1* mutants are also developmentally impaired, displaying delayed flowering time, especially when days are short and nights are long. However, gross morphological aberrations are not observed in *Arabidopsis rdr2* and *nrpd1* mutants. Maize *mop1* and *nrpd1* mutants are similarly deficient for 24-nt siRNA biogenesis, transposon silencing, and flowering but also display a high incidence of morphological abnormalities [18], [20]–[23]. The much higher transposon and retrotransposon content of the maize genome, compared to *Arabidopsis*, likely confers a greater requirement for Pol IV, RDR2/MOP1 and the siRNA-directed DNA methylation pathway to prevent transposon-induced misregulation of genes affecting morphological development.

## The Possibility of Multiple Pol IV or Pol V Sub-Types with Nuanced Functions

**Figure 1:**
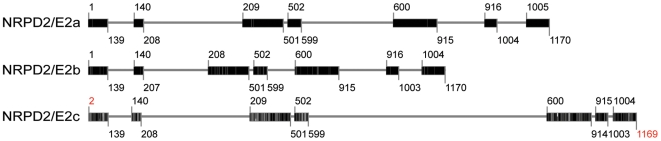
Intron-exon structures of the three *NRPD2-/NRPE2*-like genes in maize. The gene models were made using TARGeT (http://target.iplantcollaborative.org/), with the *Zea mays* NRPD2/E2a amino acid sequence obtained from the NCBI Protein database (AAY45706.1) queried against the Z. mays pseudo-genome. Exonic regions are indicated by boxes; intronic regions are marked with lines. Darker bars in exons indicate increasing similarity to the query sequence. Numbers indicate amino acid positions relative to the query sequence (NRPD2/E2a). Positions with missing amino acids relative to the query sequence are marked in red.

In the current issue, Stonaker et al. and Sidorenko et al. independently report on paramutation-impaired mutants that are defective for a maize paralog of *NRPD2/NRPE2*, the gene that encodes the second-largest subunit of both Pol IV and Pol V in *Arabidopsis*. Interestingly, maize has three such genes, with distinctive intron–exon structures (Figure 1), all of which are expressed and are presumably functional. By contrast, *Arabidopsis thaliana* has only one functional *NRPD2/NRPE2* gene. The three maize *NRPD2/NRPE2*-like genes must not be fully redundant in their functions given that Stonaker et al. and Sidorenko et al. both identified recessive mutants of the same *NRPD2/NRPE2*-like gene in their screens for paramutation-defective plants. If the different *NRPD2/NRPE2*-like genes were fully redundant, double- or triple-recessive loss-of-function mutants would have been needed to observe mutant phenotypes.

Despite the lack of full redundancy, the three maize *NRPD2/NRPE2*-like genes may be partially redundant for some Pol IV and/or Pol V functions. Evidence for this interpretation is indirect, but compelling. First, 24-nt siRNAs are severely depleted as a result of recessive loss-of-function mutations in the *NRPD2/NRPE2*-like gene that was identified in both studies; this is also true in *nrpd1* mutants, suggesting that the NRPD2/NRPE2-like protein encoded by the gene is most likely a Pol IV subunit. Both the largest and second-largest subunits of Pol IV are required for polymerase activity, as in other multi-subunit RNA polymerases, because these are the catalytic subunits [24]. Therefore, one would expect loss-of-function mutations in the *NRPD1* and *NRPD2* genes to have the same phenotypes. However, the phenotypic consequences of recessive loss of function mutations in the *NRPD2/NRPE2*-like gene that was identified (termed *NRPD2/E2a* in the Chandler lab and *NRPD2a* by the Hollick lab) are not as severe as in *nrpd1* mutants. These observations suggest that one or more of the other NRPD2/NRPE2-like proteins might also interact with NRPD1 to form Pol IV complexes with somewhat different functions, all of which are lost in an *nrpd1* mutant. This interpretation is also supported by the fact that the *Mop2-1* allele of the *NRPD2/E2a* gene identified by Sidorenko et al. is a dominant loss-of-function allele with more severe phenotypes than recessive *nrpd2/e2* loss-of-function alleles. The authors suggest that the mutated second-largest subunit in *Mop2-1* plants may assemble with other subunits to tie up NRPD1 in non-functional Pol IV complexes, thereby phenocopying *nrpd1* mutants.

## Predictions to Test, Questions to Answer

The Stonaker et al. and Sidorenko et al. papers raise a number of interesting questions and present some interpretations that will need to be further tested. First and foremost, it will be important to determine if the proteins encoded by the three *NRPD2/NRPE2*-like genes can truly serve as alternative catalytic subunits of Pol IV, Pol V, or both enzymes. Reciprocal co-immunoprecipitation studies could be one approach for revealing which of the three NRPD2/NRPE2-like proteins subunits associate with NRPD1 or NRPE1 in maize. It would be even more informative to affinity purify Pol IV or Pol V complexes containing each of the three alternative second-largest subunits and use mass spectrometry to determine the complete subunit compositions of the complexes. By this approach, *Arabidopsis* Pol IV and Pol V were recently shown to have 12 core subunits that are identical or paralogous to the 12 core subunits of Pol II [13]. For most of the smaller subunits of *Arabidopsis* Pol IV and Pol V, alternative versions of the proteins, encoded by paralogous genes, were detected in the complexes [13]. It is not yet known whether these alternative subunits are fully redundant and interchangeable in *Arabidopsis* or whether they co-associate in unique permutations within distinctive Pol IV or Pol V sub-types, but the latter is a distinct possibility. Therefore, it is possible that Pol IV and/or Pol V may come in multiple flavors, or sub-types in both maize and *Arabidopsis*, but using different sets of alternative subunits in the two species.

Another missing ingredient for interpreting the current papers is knowledge of bona fide Pol V mutant phenotypes in maize, which would best be defined by null mutations in the *NRPE1* gene encoding the Pol V largest subunit. Unfortunately, a maize *nrpe1* mutant is not yet available for comparison. In *Arabidopsis*, *nrpd1* and *nrpe1* mutants display similar losses in DNA methylation, such that DNA methylation assays do not distinguish between Pol IV and Pol V mutants. Although Pol IV is required for the biogenesis of virtually all 24-nt siRNAs in *Arabidopsis*, Pol V is also required for the biogenesis of approximately one-third of these siRNAs, is important for another one-third of the siRNAs, and is dispensable for siRNA production at the final one-third of Pol IV–dependent loci [25]. Therefore, depletion of siRNAs does not necessarily imply a Pol IV–specific function. It remains a possibility that Pol V and Pol IV might both be required for siRNA accumulation in maize, as at one-third of *Arabidopsis* loci.

The idea that Pol IV and Pol V will have the same functions in *Arabidopsis* and maize may be valid, and the three NRPD2/NRPE2-like proteins of maize may turn out to fit neatly within a Pol IV and Pol V classification due to limited overall subunit heterogeneity. But it is still early days in the study of these plant-specific RNA polymerases and their specialization in RNA-dependent silencing pathways. The possibility that maize may have an enzyme with sufficient functional and structural diversity to warrant being named Pol VI is not beyond imagination.
